# Supplementary material for: Interventions to improve mental health help-seeking attitudes, intentions and behaviors: A systematic review of recent advances
Source: Glob Ment Health (Camb). 2026 Mar 30;13:e85. doi: 10.1017/gmh.2026.10183 (PMC13125273; doi:10.1017/gmh.2026.10183)
Supplement: Bora et al. supplementary material [file S2054425126101836sup001.zip › Appendix A_Search Strategy_v01_Final.docx]

**Appendix A: Search Strategy**

**Database: Ovid Medline**

| **#** | **Query** |
| --- | --- |
| 1 | [anxiety.mp](http://anxiety.mp/). |
| 2 | Common Mental Disorder.mp. |
| 3 | CMD.mp. |
| 4 | attention deficit hyperactivity [disorder.mp](http://disorder.mp/). |
| 5 | [depression.mp](http://depression.mp/). |
| 6 | phobi*.mp. |
| 7 | schizophreni*.mp. |
| 8 | Severe Mental Illness.mp. |
| 9 | SMI.mp. |
| 10 | alzheimer*.mp. |
| 11 | [dementia.mp](http://dementia.mp/). |
| 12 | [autism.mp](http://autism.mp/). |
| 13 | down [syndrome.mp](http://syndrome.mp/). |
| 14 | addict*.mp. |
| 15 | post traumatic stress [disorder.mp](http://disorder.mp/). |
| 16 | anorexi*.mp. |
| 17 | bulimi*.mp. |
| 18 | borderline personality [disorder.mp](http://disorder.mp/). |
| 19 | self injur*.mp. |
| 20 | self [harm.mp](http://harm.mp/). |
| 21 | suicid*.mp. |
| 22 | substance [abuse.mp](http://abuse.mp/). |
| 23 | ((mental* or psychiatric) adj (health or ill* or disorder* or disabilit*)).mp. |
| 24 | ((depressive or "obsessive-compulsive" or phobic or bipolar or conduct or "impulse control" or disruptive or dissociative or eating or mood or neurocognitive or neurodevelopmental or personality or psychotic or "post traumatic" or substance or narcotic or behavio?ral or adjustment) adj3 disorder*).mp. |
| 25 | (Intellectual adj3 (disabilit* or disorder*)).mp. |
| 26 | Mental Health/ |
| 27 | exp Mental Disorders/ |
| 28 | Mentally Ill Persons/ |
| 29 | ((Service* or hospital* or care or help or treatment) adj4 utili?ation).mp. |
| 30 | ((Service* or hospital* or care or help or treatment) adj4 contact*).mp. |
| 31 | ((Service* or hospital* or care or help or treatment) adj5 access*).mp. |
| 32 | ((Service* or care or help or treatment) adj3 seek*).mp. |
| 33 | ((Service* or care or help or treatment) adj4 (using or user or usage)).mp. |
| 34 | Help-Seeking Behavior/ |
| 35 | (Randomized Controlled Trial or Controlled Clinical Trial or Pragmatic Clinical Trial or Equivalence Trial or Clinical Trial, Phase III).pt. |
| 36 | Randomized Controlled Trial/ |
| 37 | exp Randomized Controlled Trials as Topic/ |
| 38 | Controlled Clinical Trial/ |
| 39 | exp Controlled Clinical Trials as Topic/ |
| 40 | Randomization/ |
| 41 | Random Allocation/ |
| 42 | Double-Blind Method/ |
| 43 | Double Blind Procedure.mp. |
| 44 | Double-Blind Studies/ |
| 45 | Single-Blind Method/ |
| 46 | Single Blind Procedure.mp. |
| 47 | Placebos/ |
| 48 | Control Groups/ |
| 49 | (random* or sham or placebo*).ti,ab,hw,kf,kw. |
| 50 | ((singl* or doubl*) adj (blind* or dumm* or mask*)).ti,ab,hw,kf,kw. |
| 51 | ((tripl* or trebl*) adj (blind* or dumm* or mask*)).ti,ab,hw,kf,kw. |
| 52 | (control* adj3 (study or studies or trial* or group*)).ti,ab,kf,kw. |
| 53 | (Nonrandom* or non random* or non-random* or quasi-random* or quasirandom*).ti,ab,hw,kf,kw. |
| 54 | allocated.ti,ab,hw. |
| 55 | ((open label or open-label) adj5 (study or studies or trial*)).ti,ab,hw,kf,kw. |
| 56 | ((equivalence or superiority or non-inferiority or noninferiority) adj3 (study or studies or trial*)).ti,ab,hw,kf,kw. |
| 57 | (pragmatic study or pragmatic studies).ti,ab,hw,kf,kw. |
| 58 | ((pragmatic or practical) adj3 trial*).ti,ab,hw,kf,kw. |
| 59 | ((quasiexperimental or quasi-experimental) adj3 (study or studies or trial*)).ti,ab,hw,kf,kw. |
| 60 | (phase adj3 (III or "3") adj3 (study or studies or trial*)).ti,hw,kf,kw. |
| 61 | 1 or 2 or 3 or 4 or 5 or 6 or 7 or 8 or 9 or 10 or 11 or 12 or 13 or 14 or 15 or 16 or 17 or 18 or 19 or 20 or 21 or 22 or 23 or 24 or 25 or 26 or 27 or 28 |
| 62 | 29 or 30 or 31 or 32 or 33 or 34 |
| 63 | 35 or 36 or 37 or 38 or 39 or 40 or 41 or 42 or 43 or 44 or 45 or 46 or 47 or 48 or 49 or 50 or 51 or 52 or 53 or 54 or 55 or 56 or 57 or 58 or 59 or 60 |
| 64 | 61 and 62 and 63 |
| 65 | limit 64 to (english language and yr="2016 -Current") |

**Alt text:** Appendix A - Search Strategy. This appendix table provides detailed information about the search strategies employed for the databases included in the review.
