# Supplementary material for: Interventions to improve mental health help-seeking attitudes, intentions and behaviors: A systematic review of recent advances
Source: Glob Ment Health (Camb). 2026 Mar 30;13:e85. doi: 10.1017/gmh.2026.10183 (PMC13125273; doi:10.1017/gmh.2026.10183)
Supplement: Bora et al. supplementary material [file S2054425126101836sup001.zip › Appendix B_Risk of Bias_v01_Final.docx]

**Appendix B:** **Risk of bias**

B.1 Quality assessments conducted using RoB2 tool for individual RCTs.

| **Author, Year** | **Domain 1: Randomization** | **Domain 2: Deviations from Intended Interventions** | **Domain 3: Missing Outcome Data** | **Domain 4: Measurement of the outcome** | **Domain 5: Selection of the reported result** | **Domain 6: Overall Bias** |
| --- | --- | --- | --- | --- | --- | --- |
| Achtenbergh et al, 2021 | Low | Low | Low | Some concerns | Low | Some concerns |
| Aisenberg Shafran et al., 2022 | Low | Some concerns | Some concerns | Some concerns | Some concerns | Some concerns |
| Amsalem et al., 2022 | Some concerns | Low | Some concerns | High | Some concerns | Some concerns |
| Amsalem, Wall et al., 2022 | Low | Low | Low | High | High | High |
| Amsalem, Lazarov et al., 2022 | Some concerns | Low | Some concerns | Low | Some concerns | Some concerns |
| Amsalem, Wall et al., 2023 | Low | Low | Low | Some concerns | Some concerns | Some concerns |
| Amsalem, Jankowsk et al., 2023 | Low | Low | Low | Low | Some concerns | Low |
| Coleman et al., 2019 | Some concerns | Some concerns | High | High | Some concerns | High |
| Conceiç˜ao et al., 2022 | Low | Some concerns | High | High | Some concerns | High |
| Fernandez et al., 2022 | Some concerns | High | High | High | Some concerns | High |
| Finitsis wt al., 2022 | Low | Low | Low | Low | Low | Low |
| Gilgoff et al., 2022 | Low | Low | Some concerns | Low | Some concerns | Some concerns |
| Grupp Phelan et al., 2019 | Low | Low | Low | Some concerns | Some concerns | Some concerns |
| Han et al., 2023 | Low | Low | Low | Low | Low | Low |
| Hollar et al., 2020 | Some concerns | Some concerns | Low | High | Some concerns | High |
| Hollar et al., 2023 | Low | Low | Low | Some concerns | Low | Some concerns |
| Hussain et al., 2020 | Some concerns | Low | High | High | Some concerns | Some concerns |
| Hussain & Alhabash., 2020 | Some concerns | Some concerns | High | High | Some concerns | High |
| Ilgen et al., 2022 | Low | Some concerns | Low | Some concerns | High | High |
| Kerman et al., | Low | Low | Low | Low | Low | Low |
| King et al., 2018 | Low | Some concerns | Low | Low | Some concerns | Some concerns |
| King et al., 2022 | Low | Low | Low | Some concerns | Some concerns | Some concerns |
| King, 2023 | Low | Low | Low | Low | Some concerns | Low |
| Kiraz & Yildrim, 2023 | Some concerns | Low | Low | Low | Some concerns | Low |
| Kruger et al., 2023 | High | Some concerns | Some concerns | Low | Some concerns | High |
| Lustig et al., 2022 | Low | Low | Low | Some concerns | Some concerns | Some concerns |
| Martin et al., 2021 | Some concerns | Low | Some concerns | High | Some concerns | High |
| Mason et al., 2022 | Some concerns | Some concerns | Low | High | Some concerns | High |
| Milner et al,, 2018 | Some concerns | Some concerns | High | Some concerns | Some concerns | High |
| Mori et al., 2022 | Some concerns | Some concerns | Some concerns | Low | Low | Some concerns |
| Nickerson et al., 2019 | Some concerns | Low | Some concerns | High | Some concerns | Some concerns |
| Possemato et al., 2023 | Low | Low | Low | Low | Some concerns | Low |
| Scott et al., 2022 | Low | Low | Low | High | Some concerns | Some concerns |
| Sekhar et al., 2022 | Some concerns | Some concerns | Low | High | Low | High |
| Seidman et al., 2022 | Low | Some concerns | High | High | High | High |
| Shafran et al., 2019 | Some concerns | Some concerns | High | High | Some concerns | High |
| Smith et al., 2022 | Low | High | Low | Low | Some concerns | Some concerns |
| Stecker et al., 2023 | Some concerns | Low | Low | Some concerns | Some concerns | Some concerns |
| Straszewski et al., 2018 | Low | Low | Some concerns | Low | Low | Low |
| Tay, 2022 | Low | Some concerns | Low | Low | Low | Low |
| Till et al., 2023 | Low | Low | Low | Low | Low | Low |
| Tobias et al., 2021 | Low | Low | High | Low | Low | Low |
| Ugarte et al., 2023 | Low | Some concerns | High | Some concerns | Low | High |
| Wiljer et al., 2020 | Low | Low | Low | Low | Some concerns | Low |

B.2 Quality assessments conducted using RoB2 tool for cluster RCTs.

| **Author, Year** | **Domain 1a: Randomization process** | **Domain 1b: Risk of bias from timing** | **Domain 2: Deviations from intended interventions** | **Domain 3: Missing outcome data** | **Domain 4: Measurement of outcome** | **Domain 5: Selection of reported result** | **Domain 6: Overall Bias** |
| --- | --- | --- | --- | --- | --- | --- | --- |
| Calear et al., 2022 | Low | Low | Low | Some concerns | Some concerns | Low | Some concerns |
| Casañas et al., 2022 | Some concerns | Low | Low | Low | Low | Low | Low |
| Holt et al., 2017 | Some concerns | Low | Some concerns | Low | Low | Some concerns | Some concerns |
| Jordans et al., 2021 | Low | Low | Some concerns | Low | Low | Low | Low |
| Lee et al., 2023 | Low | Low | Some concerns | High | Low | Low | High |
| Link et al., 2020 | Some concerns | Low | Low | Low | Some concerns | Low | Some concerns |
| Lubman et al., 2020 | Low | Low | Some concerns | Low | High | Low | Some concerns |
| O'Dea et al., 2021 | High | High | Low | High | High | Low | High |
| Parikh et a., 2021 | Some concerns | Low | Low | Some concerns | High | Low | Some concerns |
| Reinauer 2021 | Some concerns | Low | Some concerns | Low | Low | Low | Some concerns |
| Ries 2022 | Low | Low | Some concerns | Some concerns | Low | High | High |

**Alt text:** Appendix B - Risk of Bias. This appendix presents the results of the risk of bias assessments conducted using the RoB 2 tool. (B.1) shows the assessments for individual randomized controlled trials (RCTs). (B.2) shows the assessments for cluster randomized controlled trials (cRCTs).
